# Edwards–Wilkinson depinning transition in fractional Brownian motion background

**DOI:** 10.1038/s41598-023-39191-6

**Published:** 2023-07-29

**Authors:** N. Valizadeh, H. Hamzehpour, M. Samadpour, M. N. Najafi

**Affiliations:** 1 Department of Physics, K.N. Toosi University of Technology, Tehran, 15875-4416 Iran; 2 Department of Physics, University of Mohaghegh Ardabili, P.O. Box 179, Ardabil, Iran

**Keywords:** Condensed-matter physics, Statistical physics, thermodynamics and nonlinear dynamics

## Abstract

There are various reports about the critical exponents associated with the depinning transition. In this study, we investigate how the disorder strength present in the support can account for this diversity. Specifically, we examine the depinning transition in the quenched Edwards–Wilkinson (QEW) model on a correlated square lattice, where the correlations are modeled using fractional Brownian motion (FBM) with a Hurst exponent of *H*.We identify a crossover time $T^*$ that separates the dynamics into two distinct regimes: for $T>T^*$, we observe the typical behavior of pinned surfaces, while for $T<T^*$, the behavior differs. We introduce a novel three-variable scaling function that governs the depinning transition for all considered *H* values. The associated critical exponents exhibit a continuous variation with *H*, displaying distinct behaviors for anti-correlated ($H<0.5$) and correlated ($H>0.5$) cases. The critical driving force decreases with increasing *H*, as the host medium becomes smoother for higher *H* values, facilitating fluid mobility. This fact causes the asymptotic velocity exponent $\theta$ to increase monotonically with *H*.

## Introduction

Driven interfaces have been extensively studied due to their wide range of interdisciplinary applications. Specifically, they are relevant in fluid dynamics within porous media, as demonstrated by various works such as^1–6^. Additionally, this theory also finds application in other areas such as the motion of fire fronts^7^, the evolution of cell colony fronts^8^, and the behavior of flux lines in superconductors^9^. Furthermore, its usefulness extends to nanoelectronics technology and the development of new materials^10–12^. Recently, there has been significant interest in the depinning transition of driven interfaces in quenched random media^13–15^. This phenomenon offers intriguing examples of critical dynamics, universality, and scale-free behavior in the presence of disorder and noise, frequently taking place far from equilibrium^16–21^.Fluid imbibition in porous media^22–26^, fracture crack propagation^27–30^, and magnetic flux lines in type-II superconductors^31,32^, are just a few examples of the many applications of this theory. Several universality classes (both continuum and discrete models) have been identified, such as the quenched Edwards-Wilkinson (QEW)^33,34^, KPZ^35,36^, and directed percolation depinning (DPD) class^37,38^. For a comprehensive review, refer to^38,39^. Numerous aspects of this problem have been investigated, including the impact of obstacle correlations in the host media^14^. It’s worth noting that the specifics of the disorder heavily influence this universality class.

Considering this problem from a broader viewpoint, a crucial question is how the *disordered pattern* influences the dynamics of the driven interface. Most research in this field focuses on the uncorrelated arrangement of obstacles, which can be modeled using percolation theory. However, Grassberger recently investigated the impact of dangling ends on the depinning transition. He found that interfaces that are critically pinned in isotropic random media always belong to the critical percolation universality class, making them fractal and isotropic on a large scale^40^. Despite the extensive literature on depinning phenomena, the influence of the host porous media has received relatively little attention. In particular, the impact of correlations in the quenched noise of the host system has not been extensively studied.

Several pieces of evidence suggest that velocity exponent $\theta$ lies within the range of [0.24–0.26] for the QEW model^33,41–46^. For domain walls in disordered thin films with perpendicular magnetic anisotropy in the weak disorder limit, $\theta =0.25$ and $\alpha =1.25$, which is consistent with the QEW universality class. However, for strong disorders, the exponents are dependent on the degree of disorder, as demonstrated in a similar analysis of ferrimagnetic GdFeCo thin films^47,48^. A systematic investigation of the exponents of the QEW model was conducted in^49^, where the standard exponents of the QEW were obtained. Discrepancies in the literature were attributed to non-steady relaxation. The same study reported $\alpha =0.633$ and $\theta =0.636$ for the DPD model, and $\alpha =0.63(1)$ and $\theta =0.64(5)$ for the continuum equation with a nonlinear term^37^. The link between self-organized criticality and the depinning transition of interfaces was examined in^50^. One potential explanation for the diversity observed in these phenomena is the differences in the noise correlations in various systems. Long-range correlations were studied analytically in the depinning transition of the elastic manifold in a disordered host using dynamical renormalization group techniques^51^. Long-range correlated noise is observed in various aspects of porous media, such as porosity^52–54^, diffusion^55^, and permeability^53,54,56,57^. There are multiple techniques for incorporating long-range correlated noise into a random media upon which a dynamical model can be built. Some examples of techniques that can introduce long-range correlated noise into a random media include percolation^58–60^, Ising-like disorder^58,61^, and Gaussian random Coulomb potential^14,62,63^. In these methods, the correlations exhibit power-law behavior in certain regions of the phase space. Another crucial option that has been explored in some cases is the use of fractional Brownian motion (FBM) support^64,65^. A critical example of this is geological models that incorporate the porosity and permeability of oil reservoirs as quenched long-range random variables. In these models, the correlations are typically modeled by FBM^65^.

The objective of this paper is to systematically investigate the impact of correlations in the host random media on the depinning transition. This is achieved by modeling the host media using the FBM model, with correlations determined by a Hurst exponent that controls roughness and other statistical properties. Specifically, we examine the QEW model on top of the *H*-FBM, where the depinning transition is governed by the QEW model on top of the random correlated host media (FBM). Our findings indicate that the introduction of FBM correlations causes significant changes in the properties of the depinning transition. Specifically, the depinning transition occurs at a critical driving force $F_c$, which decreases as the Hurst exponent *H* increases. Our numerical results confirm the existence of a novel form of three-parameter scaling relations near the critical driving force $F=F_c$. These scaling relations involve exponents that vary with *H*, revealing that as the Hurst exponent increases, the host media becomes softer and the fluid flow throughout the system becomes easier.

The paper is organized as follows: in the next section, we present the setup of the problem at hand and present some primitive expectations of the standard theory of driven interfaces. Our model is presented in “Two-dimensional fractional Brownian motion (2D FBM)”. The “General properties of driven interfaces” concentrates on the general properties of the driven interfaces. In “Temporal crossover and three-variable scaling for the driven interfaces in an FBM support”, we describe the scaling relations related to our model. The results are presented in “Numerical evidences for three-variable scaling”. We close the paper with a conclusion in “Concluding remarks”.

## Two-dimensional fractional Brownian motion (2D FBM)

In this brief section, we provide an explanation of FBM, which is the random host media model used in our paper, and then introduce our driven interface model. A key question that arises is about the randomness pattern in the support media, which has typically been assumed to be uncorrelated in prior literature^38,39^. In order to address this issue, we investigate the dynamics of the driven interface in a $1+1$-dimensional random media, where the scalar displacements occur in a two-dimensional media and the quenched random force in the host is correlated. To control the correlations, we utilize 2D FBM, in which the Hurst exponent is used as an input parameter in the simulations. Our study employs the QEW equation^14,38^ as the dynamical model for the interface motion. When the Hurst exponent, *H*, is equal to 0.5, there are no correlations between the increments. However, if $H>0.5$ ($H<0.5$), the samples in the system exhibit positive (negative) correlations, leading to a smoother (rougher) surface.

**Figure 1:**
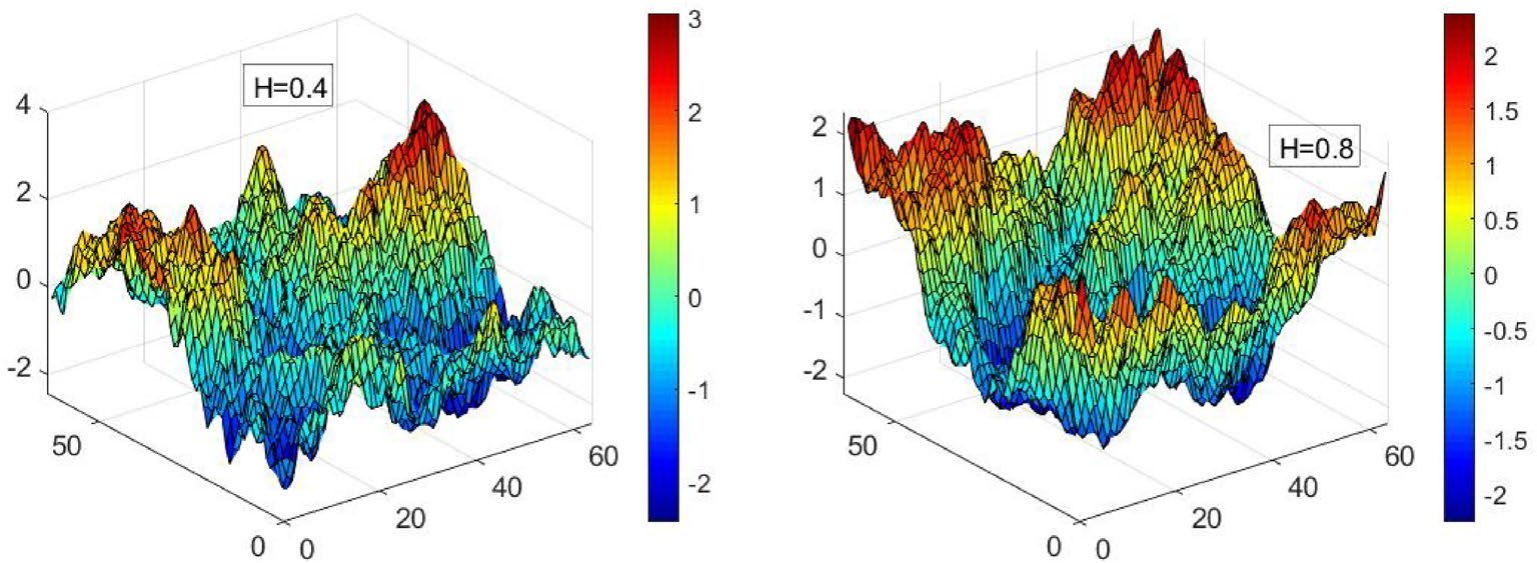
FBM disorder samples resulted from the method described in “Two-dimensional fractional Brownian motion (2D FBM)” for Hurst exponents: $H=0.4$ (left sample) and 0.8 (right sample). The vertical axis shows disorder, and the other two axes are *x*–*y* the space coordinates. The disorder samples are used for the dynamical EW model, i.e. the last term of Eq. (11). The only external parameter for constructing these samples is the Hurst exponent.

FBM is a stochastic process characterized by long-range correlations between increments and is widely used as a model for anomalous diffusion. FBM has been extensively studied in the field of mathematics, as evidenced by numerous research studies^66,67^. Moreover, this model has found widespread applications across various scientific domains and beyond. For instance, FBM has been used to investigate polymer dynamics^68,69^, analyze diffusion inside living cells^70^, study traffic patterns in electronic networks^71^, and understand the dynamics of stock markets^72^. To provide an all-encompassing overview, we will briefly summarize the key characteristics of FBM.

The correlation function of the fractional Brownian motion $B_{H}(\textbf{r})$^73^ is a stochastic process that possesses specific properties. These include $\langle B_{H}(\textbf{r})-B_{H}(\mathbf{r_{0}})\rangle =0$, and

1 $$\begin{aligned} \langle [B_{H}(\textbf{r})-B_{H}(\mathbf{r_{0}})]^{2}\rangle =|\textbf{r}-\mathbf{r_{0}}|^{2H} , \end{aligned}$$

where $\textbf{r}=(x,y)$ and $\mathbf{r_{0}}=(x_{0},y_{0})$ denote two arbitrary points, and *H* represents the Hurst exponent. We produced correlated energy landscapes in the form of FBM samples on a two-dimensional $L_x \times L_y$ lattice using the fast Fourier transformation method, which allowed us to regulate the type and intensity of correlations. To generate two-dimensional FBM, we started by creating standard Brownian motion on the $L_x\times L_y$ lattice, represented by $\zeta ({\textbf {r}})$, where ${\textbf {r}}$ denotes a point on the lattice. $\zeta ({\textbf {r}})$ is an uncorrelated random noise characterized by a Gaussian distribution, thereby satisfying the condition that:

2 $$\begin{aligned} \left\langle \zeta ({\textbf {r}})\right\rangle =0\ , \ \left\langle \zeta ({\textbf {r}})\zeta ({\textbf {r}}')\right\rangle =D\delta ^2({\textbf {r}}-{\textbf {r}}') . \end{aligned}$$

The parameter *D* represents the disorder strength. The fractional Fourier component can be expressed as:

3 $$\begin{aligned} \tilde{B}_H({\textbf {k}})=\left( k_x^{2}+k_y^{2}\right) ^{-(H+1)/2}\tilde{\zeta }({\textbf {k}}) . \end{aligned}$$

Here, ${\textbf {k}}\equiv (k_x,k_y)$ denotes the Fourier space (reciprocal lattice), and

4 $$\begin{aligned} \tilde{\zeta }({\textbf {k}})=\frac{1}{\sqrt{L_xL_y}}\sum _{{\textbf {r}}}\zeta ({\textbf {r}})e^{i{\textbf {k}}.{\textbf {r}}} , \end{aligned}$$

is the Fourier component of $\zeta ({\textbf {r}})$, and ${\textbf {r}}\equiv (x,y)$ runs over all lattice points. Given this, the FBM is given by the equation

5 $$\begin{aligned} B_H({\textbf {r}})=\frac{1}{\sqrt{L_xL_y}}\sum _{{\textbf {k}}}\tilde{B}_H({\textbf {k}})e^{-i{\textbf {k}}.{\textbf {r}}}, \end{aligned}$$

where ${\textbf {k}}$ runs over all lattice points in the reciprocal lattice. For our later use, it is convenient to compute the scaling properties of this function. From Eq. (1) one can show by inspection that

6 $$\begin{aligned} B_H(\lambda {\textbf {r}}){\mathop {=}\limits ^{d}}\lambda ^{H}B_H({\textbf {r}}). \end{aligned}$$

The symbol ${\mathop {=}\limits ^{d}}$ denotes the equality of probability measures. To comprehend this concept (refer to Appendix A), we commence with the definition of $\zeta$, which leads to

7 $$\begin{aligned} \zeta (\lambda {\textbf {r}}){\mathop {=}\limits ^{d}} \lambda ^{-1}\zeta ({\textbf {r}})\ ,\ \tilde{\zeta }(\lambda ^{-1} {\textbf {k}}){\mathop {=}\limits ^{d}} \tilde{\zeta }({\textbf {k}}), \end{aligned}$$

where the second equality is deduced simply from the Fourier analysis. This, when combined with Eq. (3) results in the following scaling relation

8 $$\begin{aligned} \tilde{B}_H(\lambda ^{-1} {\textbf {k}}){\mathop {=}\limits ^{d}} \lambda ^{H+1}\tilde{B}_H({\textbf {k}}), \end{aligned}$$

which, using the Eq. (5) gives

9 $$\begin{aligned} B_H(\lambda {\textbf {r}}){\mathop {=}\limits ^{d}}\lambda ^{H}B_H({\textbf {r}}). \end{aligned}$$

To generate a correlated energy landscape using the properties of FBM, we employed the fast Fourier transformation method. This approach allows us to regulate both the type and intensity of correlations present. Essentially, we produce random Fourier coefficients with a density specified by the desired distribution, followed by an inverse Fourier transformation to yield the energy landscape in the spatial domain. Furthermore, it is possible to express the spectral density of a two-dimensional FBM as

10 $$\begin{aligned} S({\textbf {k}})=\frac{a}{\sqrt{(k_x^{2}+k_y^{2})^{H+1}}}, \end{aligned}$$

where *a* is a constant. In this paper, we generated 2D FBM arrays of size $L_x \times L_y$ and consider it as a quenched disorder on top of which the dynamic model runs. Two FBM disorder samples are shown in Fig  1 for $H=0.4$ and $H=0.8$, from which we observe that the higher the amount of *H*, the softer the disorder sample is.

## General properties of driven interfaces

Let us consider a fluid flowing through a porous host medium with *d* dimensions (in this paper, $d=2$ is used throughout). In this dynamic process, any segment of the interface between two fluids (e.g., wet fluid and air) has the potential to adhere to a random obstacle in the porous medium, which retards the growth of the fluid cluster. When a part of the interface sticks to an obstacle, the region in its close vicinity is immediately affected, forcing that part to pass through the obstacle, resulting in a decrease in the velocity of that region. The future of the interface is determined by the interplay of two forces, namely, the random resisting force $\eta$ that stems from the obstacles, and the constant driving force *F* that facilitates the expansion of the growing cluster over the porous media. The average velocity of the fluid is expected to depend on the difference between these two forces. However it has been shown that this velocity is not a smooth function of this difference, but instead, the system undergoes a *depinning transition* between the pinned phase ($F<F_c$ in which the velocity asymptotically stops, where $F_c$ is a critical force), and the moving phase ($F>F_c$ in which the interface has a non-zero asymptotic average velocity).This means that In the pinned phase, the random resisting force is statistically more dominant than the driving force.

Numerous experiments have been conducted to observe driven interfaces, such as fluid-fluid displacement ^1,74,75^ and the process of coffee imbibition in paper towels ^76–78^. These experiments have reported varying exponents for critical driven interfaces ^38^. Additionally, a plethora of effects have been studied in this field, including 1/*f* noise in driven interfaces ^79^ and anomalous noise in driven interfaces ^2^.

The spatial coordinates are denoted by ${\textbf {r}}\equiv (x,y)$, while time is represented by *T*, with $\tilde{h}(x,T)$ being the scalar displacement field. The dynamics commence from a flat line at $y=0$, and the movement pattern of the one-dimensional interface $y=\tilde{h}(x,T)$, which serves as the boundary between the “dry” and “wet” phases, is tracked over time. Specifically, the interface responds to both the “quenched resisting noise” $\eta (x,y)$ distributed throughout space and the driving force (*F*). Our study is focused on the dynamics of a driven interface in a two-dimensional random media, which is characterized by stochastic obstacles. The positions and strengths of these obstacles are correlated and are generated using FBM, as described in the preceding section (see Fig  1). In this $1 + 1$-dimensional dynamics, the position of the interface is represented by the vertical coordinate $y = \tilde{h}(x, T )$. The QEW equation, which governs the behavior of $\tilde{h}(x,T)$, is given by:

11 $$\begin{aligned} {\partial _{T}} \tilde{h}(x,T)=F+\upsilon \partial _x^{2}\tilde{h}(x,T)+B_H(x,\tilde{h}(x,T)). \end{aligned}$$

In the given equation, $B_H(x,y)$ represents an FBM, while the surface tension is denoted by $\upsilon$, which we assume to be $\upsilon \equiv 1$. The peculiar behavior of this function can be explained using a simple scaling argument. According to Eq. (9), the scaling relations should hold at $F=F_c$. This implies that ($\tilde{h}(\lambda ^2x,\lambda T)=\lambda ^b\tilde{h}(x, T)$).

12 $$\begin{aligned} \lambda ^{b-2} {\partial _{T}} \tilde{h}(x,T)=F'_c+\lambda ^{b-2}\upsilon \partial _x^{2}\tilde{h}(x,T)+\lambda ^{2H-2}B_H(x,\tilde{h}(x,T)). \end{aligned}$$

Here, $F'_c$ represents the rescaled value of $F_c$. In the special case where $H=1$, we have $b=2$ and $F'_c=F_c$. However, for a general value of *H*, we obtain the following expression:

13 $$\begin{aligned} b=2H\ , \ F_c\rightarrow \lambda ^{2H-2}F_c, \end{aligned}$$

i.e. $F_c\rightarrow 0$ for $\lambda \rightarrow \infty$ for $H<1$. The fact that makes this problem non-trivial is the anisotropy the gives arise in the arguments of $B_H$, i.e. $B_H(x,\tilde{h})\rightarrow B_H(\lambda ^{2}x,\lambda ^{b}\tilde{h})$. Therefore, this problem needs a more detailed analysis, which is the main mission of the present paper.

The Eq. (11) is inadequate for numerical simulations, particularly when dealing with large values of F, which cause an excessive growth of $\tilde{h}$, leading to numerical instabilities. To overcome this problem, we rescale the equation and introduce a new variable $h\equiv F^{-1}\tilde{h}$. It is worth noting that after the calculations, the actual scalar displacement is given by *hF*. Therefore, we obtain the following expression:

14 $$\begin{aligned} {\partial _{T}} h(x,T)=1+\partial _x^{2}h(x,T)+B^F_H(x,h(x,T)), \end{aligned}$$

where $B^F_H(x,y)\equiv F^{-1}B_H(x,Fy)$ which is much more appropriate for the simulations. We emphasize that the real height is *hF*. For small enough driving forces the interface is shown to be in the “pinned” phase in which the interface stops, more precisely $\bar{h}(T)\equiv \frac{1}{L}\sum _{x=1}^L h(x,T)$ [where the over line represents the spatial average over parallel (*x*) coordinate $\overline{O}\equiv \frac{1}{L}\sum _i O(x=i)$, and *L* is the system size] vanishes asymptotically, while for large enough *F*s the interface is in the “moving” phase. In the vicinity of the critical driving force $F_c$ the correlation length of the system becomes the same order of the system size, and the system becomes scale-invariant resulting in scaling behaviors of the observables.

By defining the average velocity of an interface as

15 $$\begin{aligned} v(T,F,L)= \frac{\partial \left\langle \bar{h}\right\rangle }{\partial T}, \end{aligned}$$

where the $\left\langle \cdots \right\rangle$ is the ensemble average, the order parameter of the depinning transition is obtained to be

16 $$\begin{aligned} v_{\infty }\left( F,L \right) \equiv \lim _{T\rightarrow \infty } v(T,F,L). \end{aligned}$$

The condition $v_{\infty }\left( F<F_c,L \right) =0$ characterizes the pinned phase, while $v_{\infty }\left( F>F_c,L \right) >0$ corresponds to the moving phase. The QEW model is applicable when $\eta (x,y)$ is an uncorrelated quenched random noise. At $F=F_c$, the interface becomes a self-similar extended object, and its geometrical observables exhibit scaling behaviors. For instance, the interface width (roughness), which measures the degree of twisting of the growing surface of the fluid, is defined by:

17 $$\begin{aligned} w(T,F,L)^2=\left\langle \overline{\left( h(x)-\bar{h} \right) ^2} \right\rangle . \end{aligned}$$

In fact, for *all* values of *F*, two distinct regimes are identified, separated by a characteristic time scale $T_X$. Specifically, for $T\ll T_X$, the roughness *w* grows as a power-law function of time $w(L,t)\sim T^{\beta }$, where $\beta$ is the growth exponent that characterizes the roughening process. On the other hand, for $T\gg T_X$, *w* saturates to an *L*-dependent value $w_{\text {sat}}\propto L^{\alpha }$, where $\alpha$ is referred to as the roughness exponent. It should be noted that for a system in the vicinity of the critical point, $T_X\sim \xi _F^{z}$, where $\xi _F$ denotes the *correlation length*. The characteristic time scale $T_X$ scales with *L* as a power-law function, $T_X\sim L^{z}$, where the dynamic exponent *z* is given by $\frac{\alpha }{\beta }$. These relations are summarized by the following well-known scaling relation:  ^14,39,80–89^

18 $$\begin{aligned} w(T,F,L)=L^{\alpha }G_F^{(w)}\left( \frac{T}{L^{z}}\right) =T^{\frac{\alpha }{z}}{G_F^{(w)}}'\left( \frac{T}{L^{z}}\right) , \end{aligned}$$

where the functions $G_F^{(w)}(y)$ and ${G_F^{(w)}}'(y)=y^{-\frac{\alpha }{z}}G_F^{(w)}(y)$ are some *F*-dependent functions with an asymptotic behavior

19 $$\begin{aligned} G_F^{(w)}(y)=\left\{ \begin{matrix} y^{\frac{\alpha }{z}} &{} y\ll 1\\ const. &{} y\gg 1 \end{matrix} \right. \end{aligned}$$

Importantly for QEW we have $\alpha _{\text {QEW}}=0.92(4)$, $\beta _{\text {QEW}}=0.85(3)$, and $z_{\text {QEW}}=1.08(1)$.

Next, let us examine the behavior of the velocity at and around the transition point. It is a well-established fact in driven interfaces that at the depinning transition point $F=F_c$, the average velocity exhibits the following behavior^8^:

20 $$\begin{aligned} v_c(T,L)\equiv v(T,F_c,L\rightarrow \infty )\sim T^{-q}~, \end{aligned}$$

with time, while for $F<F_c$ but in the vicinity of the transition point

21 $$\begin{aligned} v(T,F,L\rightarrow \infty )\sim e^{-T/T_X}=e^{-T/\xi _{F}^z}. \end{aligned}$$

In this equation $\xi _F$ is called the *correlation length*, and *z* is the dynamical exponent as stated before. The correlation length behaves like

22 $$\begin{aligned} \xi _F\sim |F-F_c|^{-\nu }. \end{aligned}$$

Here, $\nu$ represents the correlation length exponent. In the vicinity of $F_c$, the velocity scales with the reduced force $f\equiv \frac{F-F_c}{F_c}$ in a power-law form. Specifically, we have:

23 $$\begin{aligned} v_{\infty }\left( F,L \rightarrow \infty \right) \sim f^{\theta }. \ \ \ \end{aligned}$$

Here, $\theta >0$ is known as the velocity exponent. In the vicinity of the depinning transition, certain regions of the interface are growing, while others remain pinned, forming pinning paths. The growth of the interface occurs through the propagation of these growing regions. Since the characteristic time required for this propagation is $T_X$ (i.e., the time required for correlations to propagate across the system), and the typical advancement for each movement (from one pinning site to another) is $w_{\text {sat}}$, we obtain the following expression^38^:

24 $$\begin{aligned} v_{\infty }\left( F,L \rightarrow \infty \right) \propto \frac{w_{\text {sat}}}{T_X}\propto \xi _F^{\alpha }/\xi _F^{z}\propto f^{\nu (z-\alpha )}, \end{aligned}$$

giving us the hyperscaling relation $\theta =\nu (z-\alpha )$.

A precise identification of the exponents, and the hyperscaling relations is needed to distinguish various universality classes. For example, to distinguish the QEW and QKPZ universality classes, one may start from a tilted initial configuration, and use the velocity predictions for the tilted configurations^90,91^. For the (discretized) quenched Edwards-Wilkinson (QEW) equation the critical exponents were found to be $\alpha = 1.250$ and $\theta =0.25$^41–43^, see also ^33,44,45^.

### The simulation method

In the simulations, a square lattice of size $L_x\times L_y$ is considered, where the interface grows along the *y*-axis starting from $y=0$, and the coordinates are denoted by (*x*, *y*). To solve the EW equation (14), the finite element method is utilized for both time (*T*) and space (*x*). The growth direction *h*(*x*, *T*), which is perpendicular to the *x* direction, is inherently continuous but is discretized for the purpose of simulations ($y\equiv \text {int}[h(x,T)]$). The first-order (Euler) discretization method was utilized, which exhibits sufficiently clean scaling properties (it is worth noting that the QEW model is linear) required for determining the critical exponents. Some references that demonstrate the extraction of critical exponents using this method include ^41,49,92,93^. At each point of the lattice, a quenched noise given by FBM is taken into account and inserted into Eq. (14). For generating FBM samples we use Eq. 3 with $N_x = 2^{5},2^{6},2^{7},2^{8},2^{9},2^{10}$ and $N_y = 2^{14}$ in all of our simulations. We generated over $2\times 10^4$ FBM samples and simulated the motion of one interface for each sample using Eq. (14), i.e. we have generated $2\times 10^4$ interfaces for ensemble averaging. Henceforth, we will refer to the vertical direction (i.e., the *y* axis along which the interface grows) as the time direction and the *x* axis as the space direction. Typically, we require samples that are more extensive along the time direction since the interface requires more space to attain the steady-state. In this study, we examine samples with $L_y= 2^{14}$ and $L_x\equiv L=32$, 64, 128, 256, 512, and 1024, a space step of $\Delta x=1$, and a time step of $\Delta t=0.01$. The simulations commence with a flat initial condition, and we average over $2\times 10^4$ realizations.

## Temporal crossover and three-variable scaling for the driven interfaces in an FBM support

In this section, a scaling relation involving three variables is introduced which is in agreement with our numerical outcomes for the driven interfaces in the FBM support. Like the standard theory of driven interfaces, in our model, a pinning-depinning transition is observed, which occurs at a specific critical value $F_c$ of the driving force. In general, determining $F_c$ for finite systems is often a challenge. By examining only the initial behavior of the velocity for extended periods, it can be difficult to discern what happens in the limit as $T\rightarrow \infty$ and $L\rightarrow \infty$. Furthermore, the non-uniform motion of the interface just above the threshold and the absence of a stationary regime add to the challenges in determining $F_c$. At any given moment for $F\gtrsim F_c$, the interface is composed of both pinned and unpinned regions ^39^. When the driving forces become stronger than the pinning forces in a specific area, the interface “jumps” forward but is eventually halted once more by another area with strong pinning sites. Consequently, the interface displays gradual, smooth motion punctuated by jumps, which leads to non-stationary velocities. The stationary phase is expected only for *F* values considerably greater than $F_c$ (i.e., the moving phase). A common approach to circumvent the complexities involved in determining $F_c$ is to employ data collapse analysis based on some suggested scaling relations, enabling us to obtain the critical value. In this study, we propose such a scaling relation involving three parameters and demonstrate that the numerical results are consistent with it.

### Three-variable scaling relation for the velocity

**Figure 2:**
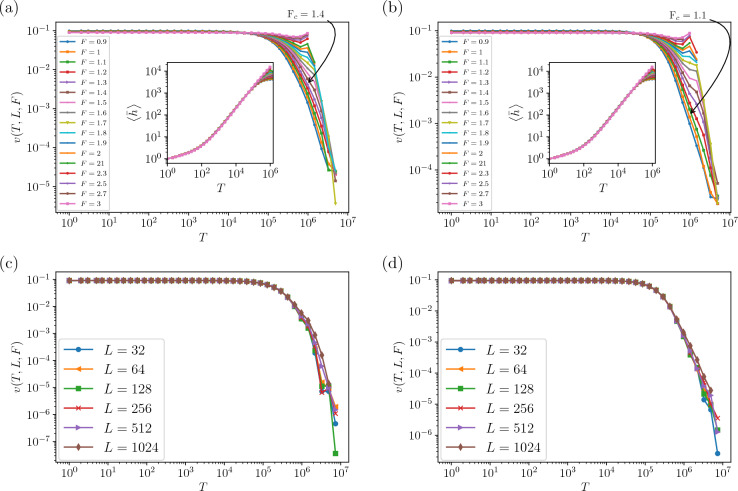
Velocity *v*(*T*, *L*, *F*) as a function of time *T* in a log-log scale for different driving forces *F* for $L = 1024$ and (**a**) $H=0.4$ and (**b**) $H=0.8$ ($F_c$ is identified in each figure). The inset shows log–log plots of the average height $\left\langle \bar{h}\right\rangle$ versus time *T*. The same plot for various *L* values is presented in for (**c**) $H=0.4$, $F=1.4$ and (**d**) $H=0.8$, $F=1.1$.

For the sake of clarity and coherence, we outline the primary results of this investigation in this section. There are two temporal regimes in our model that are separated by a *crossover* time $T^*$, at which $v_H(T,F,L)$ (the subscript *H* showing the *H*-dependence) becomes *independent of* *F* for all *H* values. In Fig. 2a, b we show the average velocity and height in terms of time *T*, from which two regimes are distinguishable. From the Fig. 2c, d, we observe that the average velocity is almost independent of *L* in the first regime, i.e. for $T<T^*$ for all *H* values. Based on this, we conjecture that the velocity fulfills the following scaling relation

25 $$\begin{aligned} v_H(T,F,L)=\left\{ \begin{matrix} v_<(T,F) &{} T< T^*\\ v^* &{} T=T^*\\ v_>(T,F,L) &{} T> T^* \end{matrix}\right. , \end{aligned}$$

all of which should have a subscript *H*, which is ignored from now on to simplify the notation, and $v^*$ is independent of *F* and *L*. Our three-variable scaling theory is based on the following asymptotic properties in terms of *f*, *L* and $t\equiv \frac{T-T^*}{T^*}$, which is presented in the following:

- A significant scaling relation pertains to the velocity at infinite time, $v_{\infty }(F,L)=\lim _{T\rightarrow \infty }v_>(T,F,L)$. It conforms to the expected behavior outlined in the standard theory of pinned interfaces, specifically Eq. (23). More precisely, this function exhibits the following scaling relation:
  26 $$\begin{aligned} v_{\infty }(F,L)&=L^{\frac{\theta }{\gamma _F}}G_{v_>}(fL^{-1/\gamma _{F}}) \nonumber \\&=f^{\theta }G_{v_>}'(fL^{-1/\gamma _{F}}), \end{aligned}$$
  where $G_{v_>}(x)$ and $G_{v_>}'(x)\equiv x^{-\theta }G_{v_>}(x)$ are scaling functions with $G_{v_>}'(x)|_{x\rightarrow 0}=\text {const}$ (corresponding to a finite *f* and $L\rightarrow \infty$, note also that $x\equiv fL^{-1/\gamma _F}$). This implies that $G_{v_>}$ satisfies
  27 $$\begin{aligned} G_{v_>}(x)=x^{\theta },\ \text {for small}\ x\ \text {values}. \end{aligned}$$
  On the other hand, one can show by inspecting Eq. (14) that for finite *L* and $f\rightarrow \infty$ ($x\rightarrow \infty$) *h* linearly grows with *T*, i.e. $h\propto T$, so that $v_{\infty }(F,L)\rightarrow \text {const}$, leading to $G_{v_>}'(x)|_{x\rightarrow \infty }=x^{-\theta }$. In this paper we use this equation for extracting $F_c$ numerically. To this end, one uses the data collapse analysis ^94^. One can extract the transition point $F_c$ using the analyticity of $G_{v_>}'$ at $f=0$, which forces
  28 $$\begin{aligned} \left. f^{-\theta }v_{\infty }(F,L)\right| _{x\rightarrow 0}=\text {const.}+a x+ \cdots \end{aligned}$$
  This shows that the transition point in the thermodynamic limit is a point at which all graphs meet each other, which is a standard method in the critical phenomena ^95^.
- Another scaling relation comes from the velocities in the vicinity of the crossover point $T^*$. In this case, the velocity fulfills the following scaling relation ($t\equiv \frac{T-T^*}{T^*}$)
  29 $$\begin{aligned} v(t\ll 1,F) =F^{\tau _t-b}G_F\left( tF^{-\tau _t}\right) . \end{aligned}$$
  Here, *b* and $\tau _t$ are two scaling exponents. We notice that this relation is independent of *L*. Now by expanding $G_F$ around $Q\equiv tF^{-\tau _t}=0$, we find
  30 $$\begin{aligned} G_F(Q)\equiv G^*+QG_{T^*}'\left( Q \right) +\text {O}(Q^{2}), \end{aligned}$$
  where $G^*\equiv \lim _{Q\rightarrow 0}G_F$. In the linear approximation, Eq. (29) gives rise to
  31 $$\begin{aligned} F^{-\tau _t+b}v(t\ll 1,F)= G^*+ s(F)t, \end{aligned}$$
  that
  32 $$\begin{aligned} s(F)\equiv \left[ \lim _{t\rightarrow 0} G_F'\left( tF^{-\tau _t} \right) \right] F^{-\tau _t}. \end{aligned}$$
  Observe that $F^{-\tau _t+b}v(t\ll 1,F)$ is independent of *F* and $t=0$, and linearly vanishes as $t\rightarrow 0$. For data collapse analysis, one may cast Eq. (31) to the following form
  33 $$\begin{aligned} t^{-1}\left[ F^{b}v(t\ll 1,F)- F^{\tau _t} G^*\right] = G_{T^*}'\left( tF^{-\tau _t} \right) . \end{aligned}$$
- The last important asymptotic behavior for the velocity is exactly at $F=F_c$, where a power-law behavior like Eq. (20) is expected. In this case the following scaling behavior for $v_c(T,L)\equiv v(T,F_c,L)$ fulfills the relation
  34 $$\begin{aligned} v_c(T,L)&= L^{-A-\frac{q_L}{\nu _T}} G_T\left( TL^{-1/\nu _T}\right) \nonumber \\&=L^{-A}T^{-q_L} G_T'\left( TL^{-1/\nu _T}\right) , \end{aligned}$$
  where $G_T(r)$ and $G_T'(r)\equiv r^{q_L}G_T(r)$ are some scaling functions with $G_{T}'(r)|_{r\rightarrow 0}=\text {const}$ ($r\equiv TL^{-1/\nu _T}$, and $q_L$ is *L*-dependent). Note that for small times ($T^*\ll T\ll T_X$, where $T_X\propto L^{1/\nu _T}$ is the time that finite size effects come to play) the above equation leads to
  35 $$\begin{aligned} v_c(T,L)\sim L^{-A}T^{-q_L}. \end{aligned}$$

### Anomalous roughness scaling relation

The roughness was analyzed in “General properties of driven interfaces”, in which the normal scaling relation was introduced. For our model, a new type of scaling is shown to work. We propose that the scaling relation reads

36 $$\begin{aligned} \begin{aligned} w(T,F,L)&=T^{\beta _w}L^{\gamma _w}G_w\left( \frac{T}{L^z} \right) \\&= L^{\alpha _w}G_w'\left( \frac{T}{L^z} \right) , \end{aligned} \end{aligned}$$

where

37 $$\begin{aligned} \alpha _w = \gamma _w+\beta _w z. \end{aligned}$$

In these equations $G_w(y)$ and $G_w'(y)\equiv y^{\beta }G_w(y)$ are new scaling functions with $G_w(y)|_{y\rightarrow 0}=const$ (one may say that the second line can be taken as the scaling relation, which is identical to the first relation in Eq. (18), but they are not the same since the universal function is different). Note that the hyperscaling relation Eq. (37) tells us that, since $\gamma _w$ has to be positive

38 $$\begin{aligned} z<\frac{\alpha _w}{\beta _w}. \end{aligned}$$

## Numerical evidences for three-variable scaling

**Figure 3:**
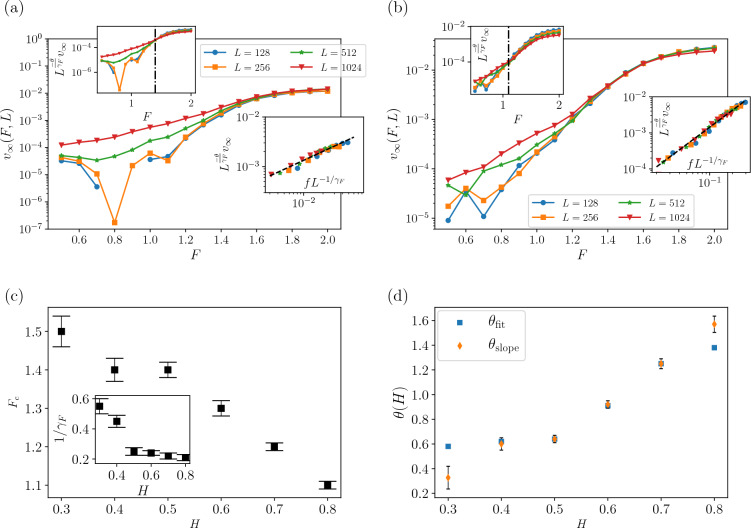
The plot of the velocity of growing interface $v_{\infty }(F,L)$ versus driving force *F*, as measured for different system sizes *L*. Upper insets: $L^{-\theta /\gamma _F}v_{\infty }$ in terms of *F* and lower insets: $L^{-\theta /\gamma _F}v_{\infty }$ in terms of $fL^{-1/\gamma _{F}}$ that is for $F>F_{c}$. For (**a**) $H=0.4$ and (**b**) $H=0.8$. (**c**) The plot $F_{c}$ in terms of *H* and we draw exponents $\theta$ and $\frac{1}{\gamma _{F}}$ in terms of *H*. (**d**) We show $\theta _{\text {fit}}=\theta$ that found with data collapse and $\theta _{\text {slope}}$, which is obtained directly by linear fitting of $v_{\infty }$-*f* graph. It is compatible with the value found for $\theta _{\text {fit}}$.

This section focuses on numerically simulating the evolution of one-dimensional rough interfaces in an FBM environment. To compute the interface’s average velocity *v*(*T*, *L*, *F*), we partitioned the total time interval into segments, with their length increasing logarithmically with time.In this study, we examined system sizes of $L=128,256,512,1024$, and the driving force was selected from the range $F\in [0.7-2]$ with a step size of 0.1. The Hurst exponents we considered ranged from $H\in [0.3-0.8]$ with an increment of 0.1. To perform the fittings and error analysis, we utilized the Curve Fitting Tool (cftool) in MATLAB. For the FBM samples, we used the method explained in “Two-dimensional fractional Brownian motion (2D FBM)”, where the Fourier transformations were directly taken.

We saw from Fig. 2 that, after a regime during which *v*(*T*, *L*, *F*) is constant, there is a cross-over time to a large time regime, based on which we proposed a three-variable scaling Eq. (25). Figure 2c, d illustrate the finite-size behavior of the average velocity, indicating that the average velocity is independent of *L* in the regime $T<T^*$, which has already led us to derive Eq. (25). The long-term behavior ($T\gg T^*$) is characterized by *F*, such that when *F* is small, *v*(*T*, *L*, *F*) rapidly diminishes to zero, indicating that the system is in the pinned phase. The moving phase, however ($F>F_c$) is identified by finite, non-zero asymptotic (final) velocities. The determination of $F_c$ is feasible upon using Eqs. (26) and (28). As depicted in Fig. 3a ($H=0.4$) and Fig. 3b ($H=0.8$), $L^{-\frac{\theta }{\nu _F}}v_{\infty }(F,L)$ becomes independent of *L* at $F=F_c$ as predicted in Eqs. (26) and (28), from which one is able to extract $F_c$. Furthermore, we extract the critical exponents (lower inset) for different Hurst exponents in Fig. 3c, d. The numerical estimations of the exponents appeared in Table 1. The fact that $\theta _{\text {fit}}$ and $\theta _{\text {slope}}$ are almost the same shows that the scaling relation Eq. (26) works properly. The exponents $\theta$ and $\gamma _F$ exhibit a sudden change in behavior at $H=0.5$, where the system transitions from a negatively correlated regime ($H<0.5$) to a positively correlated one ($H>0.5$). In the negatively correlated regime, $\theta \approx 0.6$, whereas in the positively correlated regime ($H>0.5$), $\theta$ increases with *H* and saturates at $\theta \approx 1.3$ as $H\rightarrow 1$. Note that the mean field results show that $\theta _{\text {QEW}}^{\text {MF}}=\frac{1}{3}$ which is different from our finding at $H=0.5$. Up to the knowledge of the authors, the previous numerical results have found for uniform quenched disorder distribution^33,41–46^.

**Figure 4:**
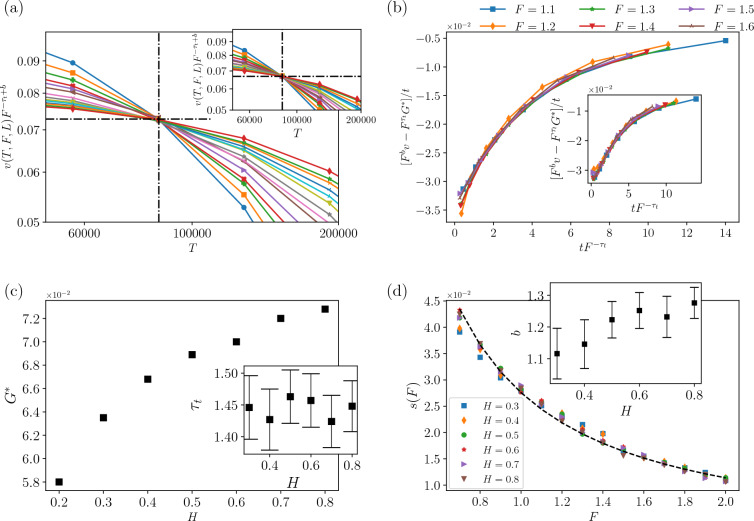
(**a**) Log–Log plots of velocity $v(T,F,L)F^{-\tau _{t}+b}$ as function of time *T* as obtained for different driving forces from up to down $F=0.7$, 0.8, 0.9, ..., 2 (with an increase of 0.1) that measured for a system size $L = 1024$ that is plotted for $H=0.8$ and inset for $H=0.4$. These data collapses show crossover point is the same ($T^{*}=85,400$) for different *H*s. (**b**) Main figure is plotted for $H=0.4$ and inset is plotted for $H=0.8$. (**c**) The plot of $G^{*}$ versus *H* and inset is $\tau _{t}$ in terms of *H*. (**d**) The dependence of *s*(*F*) versus *F* for different *H*s. The dashed line is fitting for $H=0.6$. We plotted the exponent *b* in terms of *H* in the inset.

Figure 4 is dedicated to assessing the scaling relations Eqs. (29) and (31). Figure 4a supports Eq. (29), showing that the graphs become independent of *F* at the transition point $T^*$, which is estimated to be $T^*=85,400$ for all *L* and *H* values that we examined. Figure 4a, b give us the exponents $\tau _t$ and *b* using the predicted relations in Eqs. (29), (31) and (33). Especially, Fig. 4b gives the data collapse analysis corresponding to Eq. (33). $\tau _{t}$ and *b* are presented in Fig. 4c, d terms of *H*, as well as $G^{*}$ and the slope *s*(*F*) in Eq. (32). We see that *s*(*F*) nicely fits a power-law relation with *F* as predicted in the equation. *b* is an increasing function of *H* in the anti-correlated regime ($H<0.5$, changing from $\approx$ 1.0–1.3), while it is almost fixed in the positively-correlated regime ($H>0.5$, in $\approx 1.25$). The numerical estimations are presented in Table 1.

**Figure 5:**
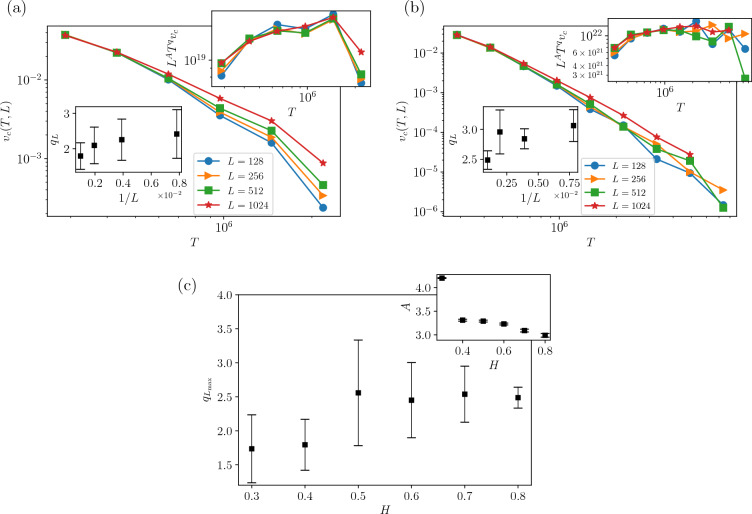
(**a**) Log–log plots of $v_{c}(T,L)$ as a function of time (*T*) at the critical driving force $F=F_{c}$ for $H=0.4$ as measured for different system sizes (*L*), as indicated. Inset: Scaling plots of the data already shown in the main plot according to Eq. (35) for different *H*s. (**b**) For $H=0.8$. (**c**) Plot $q_{L_{\textrm{max}}}$ and *A* vs *H*. The results are obtained by starting with flat interfaces and averaging over a number of 20, 000 different individual realizations and considering all the interfaces (pinned and free).

The third part of our three-variable scaling prediction is analyzed in Fig. 5 for $H=0.4$ and $H=0.8$ (a, b respectively), which assesses the relations presented in Eqs. (34) and (35). The original data is shown in the main parts, while the data collapse analysis is done in the insets. The exponent $q_L$ is sketched in terms of 1/*L* to control the finite size effects. The final estimated exponents ($q_L$ and *A*) are reported in Fig. 5c, from which we see that $q_L$ is almost robust against *H*, while *A* considerably changes.

**Figure 6:**
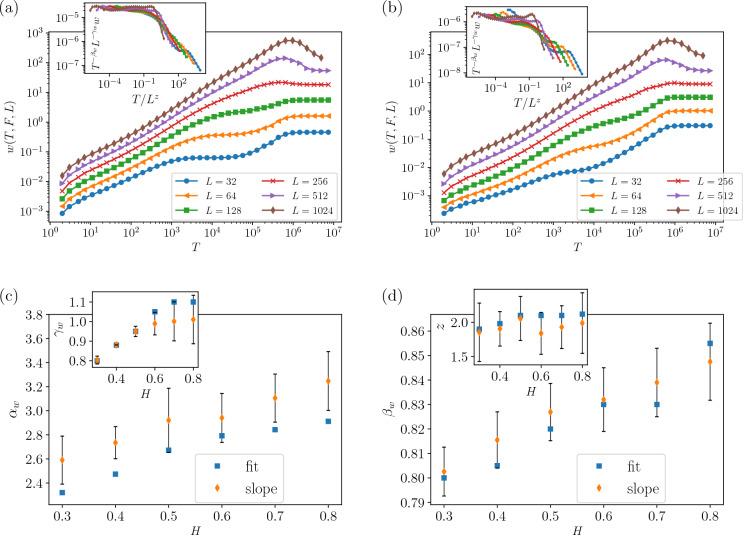
Log–Log plots of the average interface width *w* versus time *T* for different sizes *L* . From the best fit of the data in the inset for $F=F_{c}$, we obtain (**a**) For $H=0.4$ and (**b**) $H=0.8$. Exponents of roughness in terms of *H* for $F=F_{c}$, (**c**) $\alpha _w$ and $\gamma _w$, (**d**) $\beta _w$ and *z*.

To be consistent, we should examine the relations for the roughness exponent, given by Eq. (36) and the hyperscaling relation Eq. (37), which is done in Fig. 6. The exponents are extracted using the data collapse based on the Eq. (36). Figure 6 shows log-log plots of the interface width (*w*(*T*, *F*, *L*)) versus time for different system sizes and $F=Fc$. The results for $H=0.4$ ($F_c=1.4$) and $H=0.8$ ($F_c=1.1$) are shown in the figure. Generally, the analysis of $w_{sat}$ gives the required information for extracting $\alpha _{w}$, while the slope of the roughness in the early times gives information about $\beta _w$. The data collapse analysis is done in the upper insets, and the resulting exponents are presented in Fig. 6c, d. These results support the predicted scaling relation Eq. (36). The departure from the previous reports for the exponent $\alpha _w$ has roots in the fact that our scaling function is a different one.

**Table 1:** Numerical estimation of the exponents for the QEW on top of the FBM correlated lattice. The data for each set of scaling functions have been separated by a triple line, see Eqs. (26), (29), (34), and (36), which has been already shown in Figs. 3, 4, 5, and 6 respectively.

	$H=0.3$	$H=0.4$	$H=0.5$	$H=0.6$	$H=0.7$	$H=0.8$
$F_{c}$	$1.5\pm 0.04$	$1.4\pm 0.03$	$1.4\pm 0.02$	$1.3\pm 0.02$	$1.2\pm 0.01$	$1.1\pm 0.01$
$\gamma _{F}^{-1}$	$0.55\pm 0.05$	$0.45\pm 0.04$	$0.25\pm 0.025$	$0.24\pm 0.015$	$0.22\pm 0.02$	$0.21\pm 0.02$
$\theta$	$0.58\pm 0.0918$	$0.62\pm 0.05$	$0.64\pm 0.03$	$0.91\pm 0.03$	$1.25\pm 0.04$	$1.38\pm 0.066$
*b*	$1.116\pm 0.08$	$1.146\pm 0.077$	$1.223\pm 0.0575$	$1.252\pm 0.0565$	$1.232\pm 0.065$	$1.276\pm 0.049$
$\tau _{t}$	$1.446\pm 0.05$	$1.427\pm 0.048$	$1.463\pm 0.042$	$1.457\pm 0.042$	$1.424\pm 0.04$	$1.448\pm 0.04$
*q*	$1.735\pm 0.5$	$1.794\pm 0.3$	$2.558\pm 0.7$	$2.45\pm 0.5$	$2.538\pm 0.4$	$2.487\pm 0.1$
*A*	$4.2\pm 0.01$	$3.31\pm 0.02$	$3.29\pm 0.02$	$3.23\pm 0.02$	$3.09\pm 0.03$	$2.99\pm 0.04$
$\alpha _w$	$2.59\pm 0.2$	$2.73\pm 0.13$	$2.92\pm 0.26$	$2.94\pm 0.2$	$3.1\pm 0.2$	$3.247\pm 0.24$
$\beta _w$	$0.8\pm 0.01$	$0.815\pm 0.01$	$0.827\pm 0.012$	$0.832\pm 0.013$	$0.839 \pm 0.014$	$0.847\pm 0.016$
*z*	$1.855\pm 0.42$	$1.905\pm 0.25$	$2.05\pm 0.32$	$1.839 \pm 0.3$	$1.929\pm 0.31$	$1.982\pm 0.4$
$\gamma _w$	$0.8\pm 0.02$	$0.88\pm 0.01$	$0.95\pm 0.026$	$0.99\pm 0.0575$	$1\pm 0.1$	$1.01\pm 0.12$

## Concluding remarks

This study investigates the impact of support correlations on the depinning transition in two dimensions. To create the disordered host, we utilized two-dimensional fractional Brownian motion (2D FBM) and defined our Edwards–Wilkinson (EW) dynamics on top of it. The degree of correlation in FBM is determined by the Hurst exponent *H*, while the external force for the EW is denoted by *F*. We demonstrated that, as *H* increases (resulting in a smoother and softer host), the depinning transition (at which point the system enters the moving phase) occurs at lower values of *F*, indicating that the rougher the host system, the more challenging it is for the fluid to advance. The smallest $F_c$ occurs at $H=1$, where the support is much smoother and softer. Our findings also revealed that $\theta$ increases as a function of *H*, indicating that the rate of increase in asymptotic velocity with respect to *F* is higher in the moving phase, as expected. Considering the hyperscaling relation $\theta =\nu (z-\alpha )$, and noting that *z* is nearly constant and $\theta$ is an increasing function of *H*, we can conclude that the increase in $\theta$ is due to the fact that the exponent $\nu$ increases with *H*. Interestingly $q_L$ (the velocity-time scaling exponent at $F=F_c$) is almost constant in terms of *H*, while *A* (the velocity-length scaling exponent, see Eq. 35) is a decreasing function of *H*. Based on our observations, i.e. recovering three scaling relations, we proposed a three-variable scaling relation that related velocity to the time *T*, *F*, and the system size *L*. A similar scaling relation was also proposed for the roughness of the moving interface.

## Supplementary Information

Supplementary Information.

## Data Availability

The findings of this study can be supported by data that is accessible from the corresponding author upon making a reasonable request.
