# Supplementary material for: Edwards–Wilkinson depinning transition in fractional Brownian motion background
Source: Sci Rep. 2023 Jul 29;13:12300. doi: 10.1038/s41598-023-39191-6 (PMC10387108; doi:10.1038/s41598-023-39191-6)
Supplement: Supplementary file 1 — Supplementary Information. [file 41598_2023_39191_MOESM1_ESM.pdf]

0.0.1 Supplementary Material for "Edwards-Wilkinson Depinning Transition in Fractional Brownian Motion Background"

A Scaling Arguments

In this appendix, we explain the scaling properties of FBM. We start with the correlation function of FBM

$$\langle [B_H(\mathbf{r})-B_H(\mathbf{r}')]^2\rangle \propto |\mathbf{r}-\mathbf{r}'|^{2H}. \tag{1}$$

By applying a scale transformation $\mathbf{r}\to\lambda\mathbf{r}$, where λ is a non-zero positive scaling parameter, we find that FBM should scale as follows:

$$B_H(\lambda\mathbf{r}) \stackrel{d}{=} \lambda^H B_H(\mathbf{r}), \tag{2}$$

to be compatible with Eq. 1 and the main text, $\stackrel{d}{=}$ shows the equality of the probability measures. As explained in the text, to generate such a random variable we first attribute an uncorrelated Gaussian random noise over all sites of the lattice, and then re-scale the Fourier component, and then transform it back to the real space. The initial (Gaussian) white noise ($\zeta(\mathbf{r})$) has the following properties:

$$\langle\zeta(\mathbf{r})\rangle = 0\ ,\ \langle\zeta(\mathbf{r})\zeta(\mathbf{r}')\rangle = \delta^{(2)}(\mathbf{r}-\mathbf{r}'). \tag{3}$$

To proceed, we consider the scaling properties of the considered noise, which is realized in terms of the properties of the Dirac delta function, i.e.

$$\langle\zeta(\lambda\mathbf{r})\zeta(\lambda\mathbf{r}')\rangle = \lambda^{-2}\delta^{(2)}(\mathbf{r}-\mathbf{r}'), \tag{4}$$

giving rise to the scaling relation

$$\zeta(\lambda\mathbf{r}) \stackrel{d}{=} \lambda^{-1}\zeta(\mathbf{r}). \tag{5}$$

Now we need to calculate the scaling properties of the Fourier component of ζ, defined by

$$\tilde{\zeta}(\mathbf{k}) \equiv \frac{1}{L}\sum_{\mathbf{r}}\zeta(\mathbf{r})e^{-i\mathbf{k}.\mathbf{r}}, \tag{6}$$

where $\sqrt{L_xL_y}=L$ for $L_x=L_y$. Using Eq. 5, we have

$$\frac{1}{L}\sum_{\mathbf{k}}\tilde{\zeta}(\mathbf{k})e^{i\lambda\mathbf{k}.\mathbf{r}} = \lambda^{-1}\frac{1}{L}\sum_{\mathbf{k}}\tilde{\zeta}(\mathbf{k})e^{i\mathbf{k}.\mathbf{r}}. \tag{7}$$

In the thermodynamic limit we have $\sum_{\mathbf{k}}\to\left(\frac{L}{2\pi}\right)^2\int \mathrm{d}^2\mathbf{k}$. In this limit, this equation becomes

$$\frac{1}{\tilde{L}}\left(\frac{\tilde{L}}{2\pi}\right)^2\int \mathrm{d}^2\mathbf{Q}\tilde{\zeta}\left(\frac{\mathbf{Q}}{\lambda}\right)e^{i\mathbf{Q}.\mathbf{r}} = \frac{1}{L}\left(\frac{L}{2\pi}\right)^2\int \mathrm{d}^2\mathbf{k}\tilde{\zeta}(\mathbf{k})e^{i\mathbf{k}.\mathbf{r}}, \tag{8}$$

or equivalently

$$\frac{1}{\tilde{L}}\sum_{\mathbf{Q}}\tilde{\zeta}\left(\lambda^{-1}\mathbf{Q}\right)e^{i\mathbf{Q}.\mathbf{r}} = \frac{1}{L}\sum_{\mathbf{k}}\tilde{\zeta}(\mathbf{k})e^{i\mathbf{k}.\mathbf{r}}, \tag{9}$$

where

$$\mathbf{Q}\equiv\lambda\mathbf{k}\ ,\ \tilde{L}\equiv\lambda^{-1}L \tag{10}$$

are re-scaled momentum and lattice size. We note that for a periodic system $k_i=\frac{2\pi n_i}{L_i}\to Q_i\equiv\lambda\frac{2\pi n_i}{L_i}=\frac{2\pi n_i}{\tilde{L}_i}$, where $i=x,y$ are the spatial components, and $n_i=1,2,...,L_i$ are positive integers, which count the momentum, and $L_x=L_y=L$ are the linear systems sizes. This relation tells us that, in the thermodynamic limit the similarity transformation is

$$\tilde{\zeta}(\lambda^{-1}\mathbf{k}) \stackrel{d}{=} \tilde{\zeta}(\mathbf{k}). \tag{11}$$

It is worth mentioning that other definitions of the Fourier transformation lead to other scaling relations, but it does not alter the final result concerning the scaling relation for B_H. Now we use Eq. **??** to find the scaling behavior of $\tilde{B}_H$, which is

$$\begin{aligned} \tilde{B}_H(\lambda^{-1}\mathbf{Q}) &= \lambda^{H+1}\mathbf{Q}^{-H-1}\tilde{\zeta}(\lambda^{-1}\mathbf{Q}) \\ &= \lambda^{H+1}\tilde{B}_H(\mathbf{Q}). \end{aligned} \tag{12}$$

This is used to obtain the scaling behavior of B_H in the thermodynamic limit as follows

$$\begin{aligned} B_H(\lambda\mathbf{r}) &= \frac{1}{L}\left(\frac{L}{2\pi}\right)^2 \int d^2\mathbf{k}\tilde{B}_H(\mathbf{k})e^{i\lambda\mathbf{k}.\mathbf{r}} \\ &= \frac{\lambda^{-1}}{\tilde{L}}\left(\frac{\tilde{L}}{2\pi}\right)^2 \int d^2\mathbf{Q}\tilde{B_H}(\lambda^{-1}\mathbf{Q})e^{i\mathbf{Q}.\mathbf{r}} \\ &= \lambda^H\left(\frac{1}{\tilde{L}}\sum_{\mathbf{Q}}\tilde{B_H}(\mathbf{Q})e^{i\mathbf{Q}.\mathbf{r}}\right) \\ &= \lambda^H B_H(\mathbf{r}). \end{aligned} \tag{13}$$

which is the same as Eq.2.
